# Genomic Data Quality Impacts Automated Detection of Lateral Gene Transfer in Fungi

**DOI:** 10.1534/g3.116.038448

**Published:** 2017-02-23

**Authors:** Pierre-Yves Dupont, Murray P. Cox

**Affiliations:** Statistics and Bioinformatics Group, Institute of Fundamental Sciences, Massey University, Palmerston North 4442, New Zealand; the Bio-Protection Research Centre, Massey University, Palmerston North 4442, New Zealand

**Keywords:** lateral gene transfer, horizontal gene transfer, automated detection methods, comparative genomics, eukaryotes, fungi

## Abstract

Lateral gene transfer (LGT, also known as horizontal gene transfer), an atypical mechanism of transferring genes between species, has almost become the default explanation for genes that display an unexpected composition or phylogeny. Numerous methods of detecting LGT events all rely on two fundamental strategies: primary structure composition or gene tree/species tree comparisons. Discouragingly, the results of these different approaches rarely coincide. With the wealth of genome data now available, detection of laterally transferred genes is increasingly being attempted in large uncurated eukaryotic datasets. However, detection methods depend greatly on the quality of the underlying genomic data, which are typically complex for eukaryotes. Furthermore, given the automated nature of genomic data collection, it is typically impractical to manually verify all protein or gene models, orthology predictions, and multiple sequence alignments, requiring researchers to accept a substantial margin of error in their datasets. Using a test case comprising plant-associated genomes across the fungal kingdom, this study reveals that composition- and phylogeny-based methods have little statistical power to detect laterally transferred genes. In particular, phylogenetic methods reveal extreme levels of topological variation in fungal gene trees, the vast majority of which show departures from the canonical species tree. Therefore, it is inherently challenging to detect LGT events in typical eukaryotic genomes. This finding is in striking contrast to the large number of claims for laterally transferred genes in eukaryotic species that routinely appear in the literature, and questions how many of these proposed examples are statistically well supported.

LGT (also known as horizontal gene transfer) is an atypical mechanism of transmitting genes, not from one generation to the next within a single species but rather horizontally between different species (Gogarten and Townsend 2005; Keeling and Palmer 2008). Although a less common mode of inheritance, this process has been known since as early as 1969, when mutations in the galactose operon of *Escherichia coli* were found to be caused by the insertion of mobile DNA elements (Shapiro 1969). Today, substantial evidence has accumulated for LGT in prokaryotes, and LGT has been shown to impose a strong evolutionary force on prokaryotic genomes, including through the transfer of pathogenicity-related genes (Gyles and Boerlin 2014; Kay *et al.* 2002; Furuya and Lowy 2006; Ochman *et al.* 2000; Martínez 2008). Importantly, however, other studies suggest that the role of LGT in prokaryotes may be overrated (Galtier 2007). More recent studies have also revealed evidence for LGT in eukaryotic genomes (Alsmark *et al.* 2009; Nikolaidis *et al.* 2014; Richards *et al.* 2009; Soanes and Richards 2014), and in some cases even between species from different kingdoms or domains (Marcet-Houben and Gabaldón 2010; Liu *et al.* 2011; Monier *et al.* 2009; Richards *et al.* 2006; Brown 2003). This is despite earlier skepticism about the presence or extent of LGT in eukaryotes, likely due to a poor understanding of the molecular mechanisms involved (Sprague 1991; Syvanen 1994; Keeling and Palmer 2008). Nevertheless, the extent of LGT in eukaryotes is unknown, and questions are increasingly being raised over whether it has been overestimated (Danchin 2016). LGT in eukaryotes has been particularly well studied in fungi (Rosewich and Kistler 2000; Fitzpatrick 2012; Szöllősi *et al.* 2015; Khaldi *et al.* 2008; Moran *et al.* 2012; Ma *et al.* 2010), with many examples of LGT described between fungi and their plant hosts (Nikolaidis *et al.* 2014; Richards *et al.* 2009; Soanes and Richards 2014). While most early LGT studies focused on only one or a few genes, typically relying on extensive manual annotation, the wealth of genomic data now available is making whole-genome analyses of potential LGT events possible (Mallet *et al.* 2010; Wisecaver *et al.* 2014; Cheeseman *et al.* 2014; Ku *et al.* 2015). These analyses rely on two different classes of methods: composition- and phylogeny-based approaches.

Composition-based methods (Cortez *et al.* 2009; Menigaud *et al.* 2012; Jaron *et al.* 2014; Rogul *et al.* 1965; Lawrence and Ochman 1997; Nakamura *et al.* 2004; Rosewich and Kistler 2000; Marcet-Houben and Gabaldón 2010) focus on patterns in the primary structure of genes and genome sequences, and aim to find genes or genomic regions with composition patterns that differ significantly from the rest of the genome. These composition differences are often then attributed to an LGT event. A key advantage of these methods is that they do not require additional information about other organisms outside the study species, including the source species of the LGT event. However, where this extra information is available, the composition of the potentially transferred gene can be compared with the composition of other genomes to infer a putative origin of the laterally transferred gene (Menigaud *et al.* 2012). The key disadvantage of these methods is that they are sensitive to changes within the genome caused by common evolutionary features other than LGT, such as repetitive elements, AT- or GC-rich isochores, and pathogenicity or symbiosis islands, all of which display different compositions to the rest of the genome. In contrast, phylogeny-based methods (Weyenberg *et al.* 2014; Abby *et al.* 2010; Zhaxybayeva 2009; Poptsova 2009) are generally thought to be more accurate, especially for detecting transfers between closely related species (Koski *et al.* 2001; Poptsova and Gogarten 2007; Poptsova 2009). However, these methods are much more computationally intensive because they require orthology predictions, sequence alignments, and phylogeny reconstructions. They work by detecting a gene phylogeny that is statistically different from either the species phylogeny and/or the majority of other gene phylogenies. Some use a scoring system to rate phylogenetic events and employ a parsimony analysis to infer LGT *vs.* gene loss/gene duplication (Bansal *et al.* 2012; Stolzer *et al.* 2012). Phylogeny-based methods are highly dependent on the quality of the gene models, multiple sequence alignments, and phylogeny reconstruction (Beiko and Ragan 2009). Unfortunately, the results of different composition- and phylogeny-based methods generally do not overlap (Ragan 2001), leading to growing suspicion about the accuracy of these results (Than *et al.* 2007; Danchin 2016), especially since most LGT events could potentially be explained by other molecular mechanisms (Morel *et al.* 2015). However, both classes of methods can at least be used to generate hints about potential LGT cases, which might then be confirmed using extensive manual annotation in further studies (Ambrose *et al.* 2014; Cheeseman *et al.* 2014; Brown 2003).

To exchange genetic material, two species must live close by, often in the same environmental niche. Therefore, in this study, we focus on possible exchanges between plant-associated fungi using the well-studied beneficial fungal endophyte *Epichloë festucae* as a reference point (Dupont *et al.* 2015; Eaton *et al.* 2015; Schardl *et al.* 2013). Plant-associated fungi frequently cooccur, have likely physically interacted over evolutionary timescales, and therefore seem like reasonable candidates to have exchanged genes via LGT. Importantly, we use genome-scale data typical of that available today, with all its various faults from automated genome assembly, gene calling, and gene annotation. We compare both composition- and phylogeny-based methods for their ability to detect novel and proposed LGT events in fungi, and reveal new statistical insight into the pitfalls of these methodologies.

## Materials and Methods

### Data description

*E. festucae* gene models were downloaded from the University of Kentucky Endophyte Database (Schardl *et al.* 2013). Version 6 of the EfM3 protein and gene (transcript) models were used. Sequences for all other fungal and oomycete species used in this analysis were downloaded from the JGI MycoCosm database (Grigoriev *et al.* 2014). The species were chosen to avoid genus redundancy. Lower quality genomes were excluded based on sequencing read coverage, number of scaffolds, and sizes of the longest scaffolds, with the exception of the endophytes, which were all retained. These species include: symbiotic endophytic fungi [*Daldinia eschscholtzii*, *Rhodotorula graminis* (Firrincieli *et al.* 2015), *Xylona heveae* (Gazis *et al.* 2016)], mycorrhizal fungi [*Cenococcum geophilum*, *Choiromyces venosus*, *Cortinarius glaucopus*, *Gyrodon lividus*, *Hebeloma cylindrosporum* (Kohler *et al.* 2015; Dore *et al.* 2015), *Laccaria bicolor* (Martin *et al.* 2008), *Meliniomyces bicolor* (Grelet *et al.* 2009), *Oidiodendron maius* (Kohler *et al.* 2015), *Paxillus involutus* (Kohler *et al.* 2015), *Pisolithus tinctorius* (Kohler *et al.* 2015), *Terfezia boudieri*, *Tuber melanosporum* (Martin *et al.* 2010), and *Wilcoxina mikolae*)] and plant pathogens [*Bipolaris sorokiniana* (Ohm *et al.* 2012; Condon *et al.* 2013), *Blumeria graminis* (Spanu *et al.* 2010), *Botryosphaeria dothidea*, *Botrytis cinerea* (Staats and van Kan 2012; Amselem *et al.* 2011), *Cercospora zeae-maydis*, *Colletotrichum graminicola* (O’Connell *et al.* 2012), *Cronartium quercuum*, *Didymella exigua*, *Fomitiporia mediterranea* (Floudas *et al.* 2012), *Fusarium graminearum* (Cuomo *et al.* 2007), *Leptosphaeria maculans* (Rouxel *et al.* 2011), *Magnaporthe oryzae* (Dean *et al.* 2005), *Mixia osmundae* (Toome *et al.* 2014), *Passalora fulva* (de Wit *et al.* 2012; Ohm *et al.* 2012), *Phaeosphaeria nodorum* (Hane *et al.* 2007), *Sclerotinia sclerotiorum* (Amselem *et al.* 2011), *Setosphaeria turcica* (Ohm *et al.* 2012; Condon *et al.* 2013), *Sporisorium reilianum* (Schirawski *et al.* 2010), *Verticillium alfalfae* (Klosterman *et al.* 2011), and *Zopfia rhizophila*]. Transcript sequences were downloaded for all species, also from the JGI database. Protein models for *Phytophthora sojae* (Tyler *et al.* 2006), an oomycete, were used as outgroups to root the gene trees. *Pseudomonas fluorescens* sequences were downloaded from the NCBI Gene database (Redondo-Nieto *et al.* 2012).

### Orthology prediction

Gene orthologies were predicted using the Reciprocal Best Blast Hit (RBBH) method because it has been shown to have the lowest false-positive error rate (Salichos and Rokas 2011; Huerta-Cepas *et al.* 2007). Transcripts from the EfM3 gene models were used for *E. festucae*, the gene models from *Ps. fluorescens* were downloaded from the NCBI Gene database, and all the other gene models were retrieved from the JGI database. A blastn database was built for each of the comparison species, including *E. festucae*, using transcript sequences (excluding UTRs). The best blastn hit (*E*-value ≤ 1 × 10^−5^) was recovered for each gene in each species. Blastn was used to avoid problems with misannotated intron–exon boundaries. By definition, to be accepted as a reciprocal best blast hit, if gene G1 from one species is the closest hit of gene G2 in another species, the same gene G2 must be the closest blast hit of G1.

### Gene composition

The percentage of GC content (GC) and the percentage of GC on the third base of each codon (GC3) were computed on the coding sequences with a custom Python script. The same script performed the computation of the Codon Adaptation Index (CAI) using the formula of Sharp and Li (1987). The effective number of codons (ENC), a measure of codon usage bias that has received attention more recently (Sun *et al.* 2013), was computed using the method of Wright (1990).

### Phylogenetic analysis pipeline

For the protein phylogenies, the sequences of each orthology group were aligned using MAFFT v7.058b (Katoh *et al.* 2002; Katoh and Standley 2013) with the set of parameters *linsi*. Automatic trimming of the alignments was then performed using trimAl v1.2rev59 (Capella-Gutierrez *et al.* 2009). The phylogeny was built using PhyML v20120412 (Guindon *et al.* 2010) using the model LG (Le and Gascuel 2008) with 100 bootstrap replicates. The species tree was reconstructed using the Subtree Prune-and-Regraft distance (SPR) supertree method (v1.2.0) (Whidden *et al.* 2014) using all 1768 genes that had predicted orthology with *P. sojae*, the oomycete outgroup used to root the tree. A bootstrap test was performed to assess the robustness of the tree. A sample of trees was taken with replacement from the initial gene tree population and a SPR supertree was generated for each sample. This procedure was repeated 500 times. The bootstrap values of each branch correspond to the percentage of trees containing the given branch.

To infer potential LGT events, the maximum likelihood value reported by PhyML for each gene tree was compared to the maximum likelihood value of the phylogeny for the same gene with its topology constrained by the species tree topology.

### Phylogenetic-based LGT prediction methods

Three different software packages were used, all based on the evaluation of the most parsimonious Deletion Transfer Loss (DTL) scenario: Ranger-DTL v1.0 (Bansal *et al.* 2012), Notung v2.8.1.7 (Chen *et al.* 2000; Stolzer *et al.* 2012) and ecceTERA v1.2.4 (Jacox *et al.* 2016). These methods use scores for each of the three possible events: that is, deletion, transfer, and loss. The default scores were used for deletion and loss, and a range of scores were trialed for transfer, from 3 (the default value) to 30. All three methods were run on all trees containing *P. sojae* as an outgroup.

### Computation of tree distances

Only bipartitions with a bootstrap score ≥75% were used for the distance comparison. The tree distances were computed using the Robinson and Foulds (RF) algorithm (Robinson and Foulds 1981) using the normalized RF distance implemented in the ETE toolkit for Python (v2.3.7) (Huerta-Cepas *et al.* 2010). To compute phylogenetic distances between two trees, both were rooted using the same outgroup species. The bootstrap cut-off introduced polytomies in a small number of trees, which were resolved using the automatic method provided by the ETE toolkit.

### E. festucae gene clustering

The transcript sequences of *E. festucae* were clustered using the Mean Shift algorithm (Comaniciu and Meer 2002) implemented in the *scikit-learn* (v0.16.1) library for Python (Pedregosa *et al.* 2011) based on their GC, GC3, CAI, ENC, and tetranucleotide frequencies. The bandwidth was automatically determined using the method *estimate_bandwidth* available in *scikit-learn*. As the Mean Shift algorithm is sensitive to the shape of the data distributions (*i.e.*, it was built to discover “blobs”), another clustering algorithm from *scikit-learn*, DBSCAN (Ester *et al.* 1996), was also used. This algorithm considers clusters as areas of high density separated by areas of low density.

### Data availability

All data are freely available from public databases. Accession numbers are indicated in Supplemental Material, Table S1 in File S1. 

## Results

### Study system

**Figure 1:**
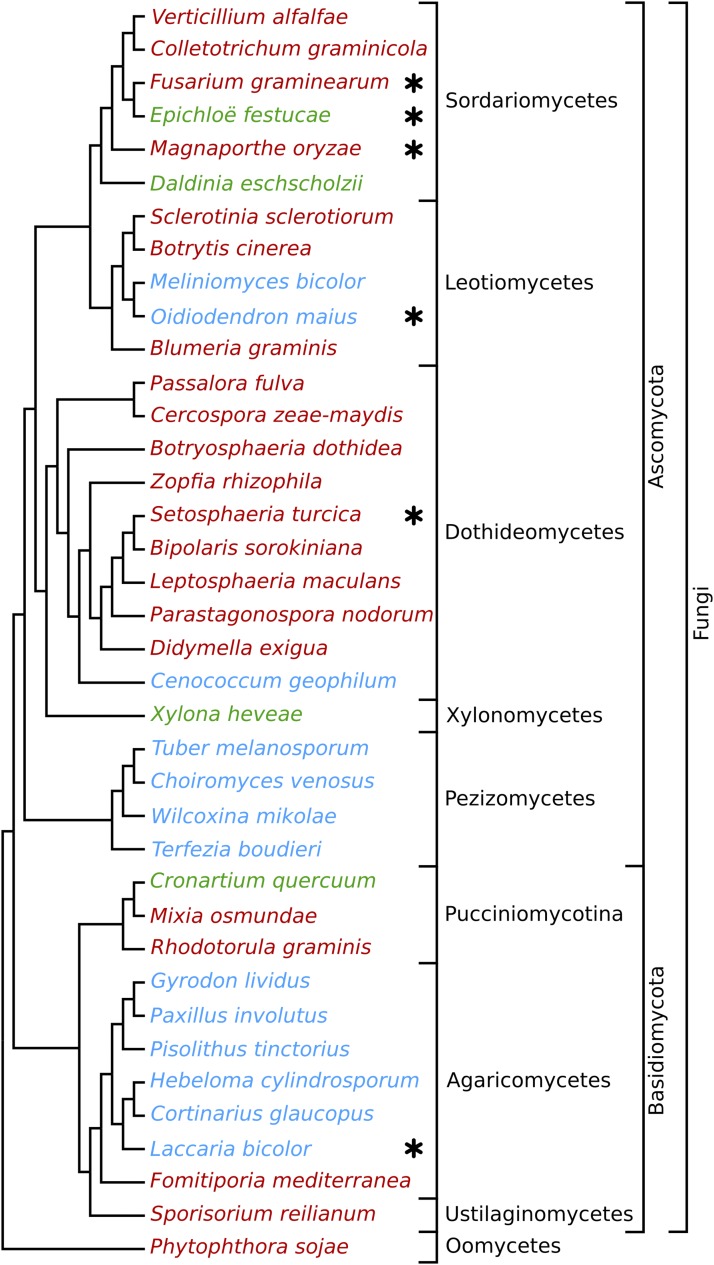
Cladogram of the plant-associated fungal species considered in this study. This tree represents the phylogenetic relationships between 37 plant-associated fungal species that span the fungal tree of life, together with an oomycete outgroup. It contains seven classes of fungi (Sordariomycetes, Leotimycetes, Dothideomycetes, Xylonomycetes, Pezizomycetes, Agaricomycetes, and Ustilagomycetes) and one Oomycetes species. Subphylum Pucciniomycotina represents three more classes of fungi: Microbotryomycetes (*R. graminis*), Mixiomycetes (*Mi. osmundae*), and Pucciniomycetes (*C. quercuum*). It covers the two main fungal phyla: Basidiomycota and Ascomycota. Our reference species, *E. festucae*, belongs to the same order (Hypocreales) as its closest neighbor in the tree, *F*. *graminearum*. The Oomycete *P. sojae* is a nonfungal organism used as an outgroup in the phylogeny reconstruction. Pathogenic species are represented in red, mycorrhizas in blue, and endophytes in green. Asterisks indicate the species at laddered evolutionary distances that are compared to *E. festucae* in Figure 4.

While claims of LGT are common in the literature (Gogarten and Townsend 2005; Keeling and Palmer 2008; Shapiro 1969; Gyles and Boerlin 2014; Kay *et al.* 2002; Furuya and Lowy 2006; Ochman *et al.* 2000; Sprague 1991; Syvanen 1994; Rosewich and Kistler 2000; Fitzpatrick *et al.* 2008; Marcet-Houben and Gabaldón 2010), comprehensive studies suggest that the power of automated methods to detect LGT events remain limited (Ragan 2001; Than *et al.* 2007; Danchin 2016). This study aims to investigate whether it is possible to predict LGT events accurately in eukaryotic systems, specifically for real genomic data from fungi, given all its associated sources of noise and error. This study focused on protein-coding genes from 37 fungal species (Table S1 in File S1) that form associations with plants (20 pathogens, 13 mycorrhiza, and four endophytes), plus one pathogenic oomycete species, *P. sojae*, used as an outgroup. The aim was to focus on species that live in a common environmental niche (the plant host), thus increasing the possibility that LGT events may have occurred between them. As is typical in modern genomic research, the genes and coding sequences studied here have mostly been automatically predicted, with little to no manual curation. Orthology relationships were determined using the RBBH method, which has been shown to offer a good balance between sensitivity and specificity in contrast to more computationally intensive algorithms (Salichos and Rokas 2011; Huerta-Cepas *et al.* 2007). The species tree presented in this study (Figure 1) was reconstructed using the SPR supertree method (Whidden *et al.* 2014) from all gene trees that included an outgroup sequence from *P. sojae* (*n* = 1768). Importantly, this species tree exactly matches previously published phylogenies (Fitzpatrick *et al.* 2006; Wang *et al.* 2009; Brown 2003; James *et al.* 2006; Liu *et al.* 2006) and is also consistent with the traditional fungal taxonomy available on the NCBI taxonomy database.

For many analyses, it is useful to view LGT from the perspective of a reference species. In this study, *E. festucae*, a natural symbiotic endophyte of the cool season grass *Festuca rubra*, was used (Schardl 2001). This organism is very well studied and used extensively as a model system to explore fungal–plant interactions (Schardl *et al.* 2013; Tanaka *et al.* 2006; Christensen *et al.* 2008; Dupont *et al.* 2015; Eaton *et al.* 2015). Because *E. festucae* is the primary model organism in our laboratory, we understand its genes and genome particularly well, which has benefited the interpretation of several analyses presented here. Five other fungal species were chosen as secondary reference points based on their laddered evolutionary distances from *E. festucae*. From the closest to the furthest, these species are *F. graminearum*, *O. maius*, *M. oryzae*, *S. turcica*, and *L. bicolor* (Figure 1).

### Assessment of previously described LGT event in E. festucae

The *fitD* gene, which encodes an insect toxin, has been reported to be a putatively laterally transferred gene from *Pseudomonas* bacteria to *Epichloë* endophytes (Ambrose *et al.* 2014). A blast and HMMER search on the UniProtKB database shows that this gene is also present in *Phlebiopsis gigantea*, a fungus from the Agaricomycetes class that is phylogenetically very distant from *E. festucae* with a much higher *E*-value than the *Pseudomonas* sequences. It is also found in *Aspergillus flavus* (Eurotiomycetes), in different *Metarhizium* species (Sordariomycetes), and in *Neonectria ditissima* (Sordariomycetes), all with significant *E*-values (< 1 × 10^−12^). It is important to consider that *fitD* may not have been directly transferred from bacteria into *Epichloë* due to its presence in many phylogenetically distant fungal clades. It is interesting to note that the closest fungal *fitD* hit is from *Ph. gigantea*, which is phylogenetically more distant from *E. festucae* than the other fungal hits, suggesting that *fitD* may have been transferred into *Epichloë* from *Ph. gigantea* rather than directly from bacteria. Indeed, it is possible that *fitD* was lost in most fungal species and may not have been laterally transferred at all. Nevertheless, *fitD* is included in many analyses here as a reference point, as it is the only LGT gene claimed to date in an *Epichloë* species.

### Composition-based methods

Composition-based methods are used to find genes whose sequence compositions differ from the rest of the genome. They do not require any knowledge about the phylogeny of the studied organism, and have a long history of use for characterizing potential LGT events (Rogul *et al.* 1965), especially in prokaryotes (Lawrence and Ochman 1997; Garcia-Vallve *et al.* 2000; Nakamura *et al.* 2004) and more recently in fungi (Rosewich and Kistler 2000; Fitzpatrick 2012; Marcet-Houben and Gabaldón 2010; Jaron *et al.* 2014). Our study aims to evaluate the efficiency of composition-based methods for detecting LGT in fungal genomes.

**Figure 2:**
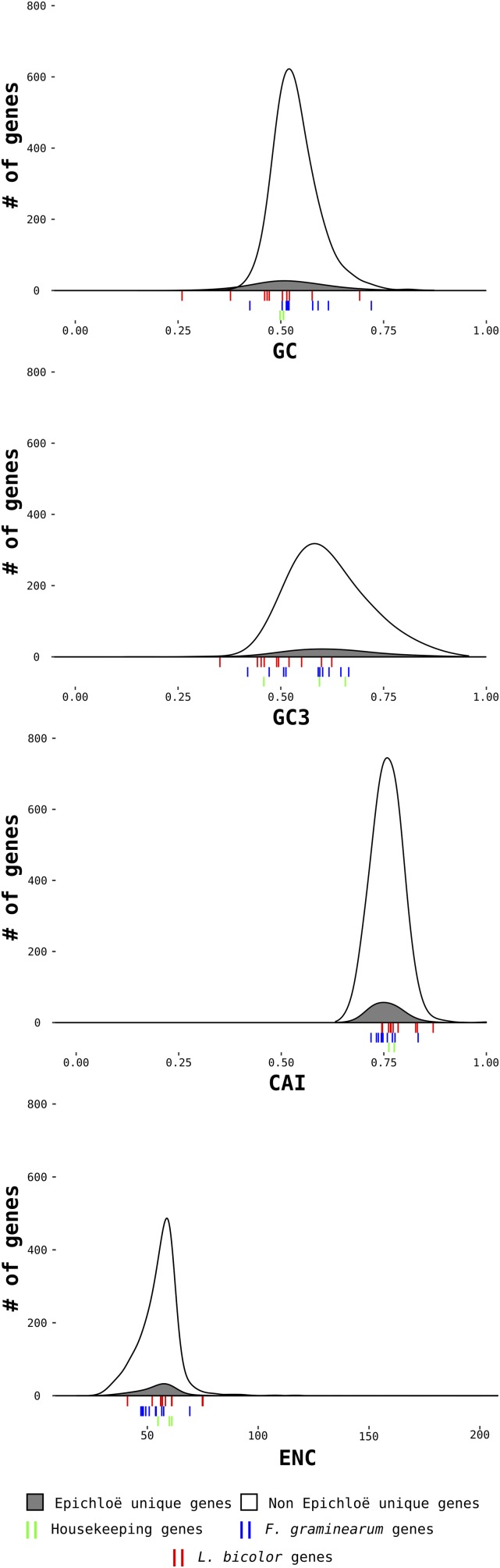
Distributions of CAI, ENC, GC, and GC3 in *E. festucae* coding sequences. Genes present only in *Epichloë* species (potentially not real genes or secondary metabolism genes with fast evolution, for example) are shaded gray. All other genes are shown in white. The values for three housekeeping genes (tefA, tuba, and actA), often used for reconstructing phylogenies and employed here as examples of putatively nonlaterally transferred genes, are shown by green bars under each graph. The values for 10 randomly drawn genes from *F. graminearum* and *L. bicolor* are represented, respectively, by blue and red bars. All graphs have the same ordinate axes. CG, GC3, and CAI are plotted using the same abscissa axes. actA, actin; CAI, codon adaptation index; ENC, effective number of codons; GC, GC content; GC3, GC content on the third position of the codons; tefA, translational elongation factor 1α; tubA, α-tubulin gene.

Four commonly used metrics were employed to characterize the coding sequences of *E. festucae*: GC, GC3, the CAI, and the ENC. For GC, GC3, and CAI, metrics can vary between 0 and 1. The values of these four metrics in *E. festucae* are presented in Figure 2. Summary statistics of these metrics are shown in Table S2 in File S1. In order to be detected, LGT genes must display a composition value that is significantly different from the rest of the gene set of the organism, which will result in the gene falling into one of the two tails of the distribution curves. The wider the genomic distribution (as for GC3; range = 0.84; Table S2 in File S1), the harder it is to detect an LGT event amid the noise of the distribution. The composition values for genes found only in *Epichloë* spp. and not in other fungal species are plotted separately in gray (Figure 2). Genes from species absent from the NCBI nonredundant database also fall into this category. It was important to separate these “orphan” genes from the rest of the gene set as the chance of them being misannotated, given their lack of orthologs, is much higher. However, as the curves for *Epichloë*-unique and -nonunique genes overlap, it appears that the unique genes do not have clear differences in composition compared to the rest of the genome. LGT events were simulated in Figure 2 by comparing composition values for 10 randomly chosen genes from *L. bicolor* and *F. graminearum* with the distributions of *E. festucae* genes. Strikingly, it is not possible to distinguish most of the gene differences, even for genes from *L. bicolor*, the most distant species. The values for the *fitD* gene (GC = 0.53, GC3 = 0.49, CAI = 0.79, and ENC = 59) are also not different from the rest of the *E. festucae* genes. To further investigate the potential ability of these metrics to predict LGT events, outlier genes that fell within the peripheral 2.5% of each side of the distribution curves were analyzed manually. Blast analysis against the nonredundant NCBI database was used to examine homology to genes from other species. In many cases, homologs were identified in other closely related species. When a protein function was available for the homologous sequences, no obvious reason for considering the gene as a potential LGT event could be found (*e.g.*, many were key housekeeping genes involved in primary metabolism with orthologs in closely related species), although this obviously does not allow us to dismiss the possibility of an LGT event completely.

**Figure 3:**
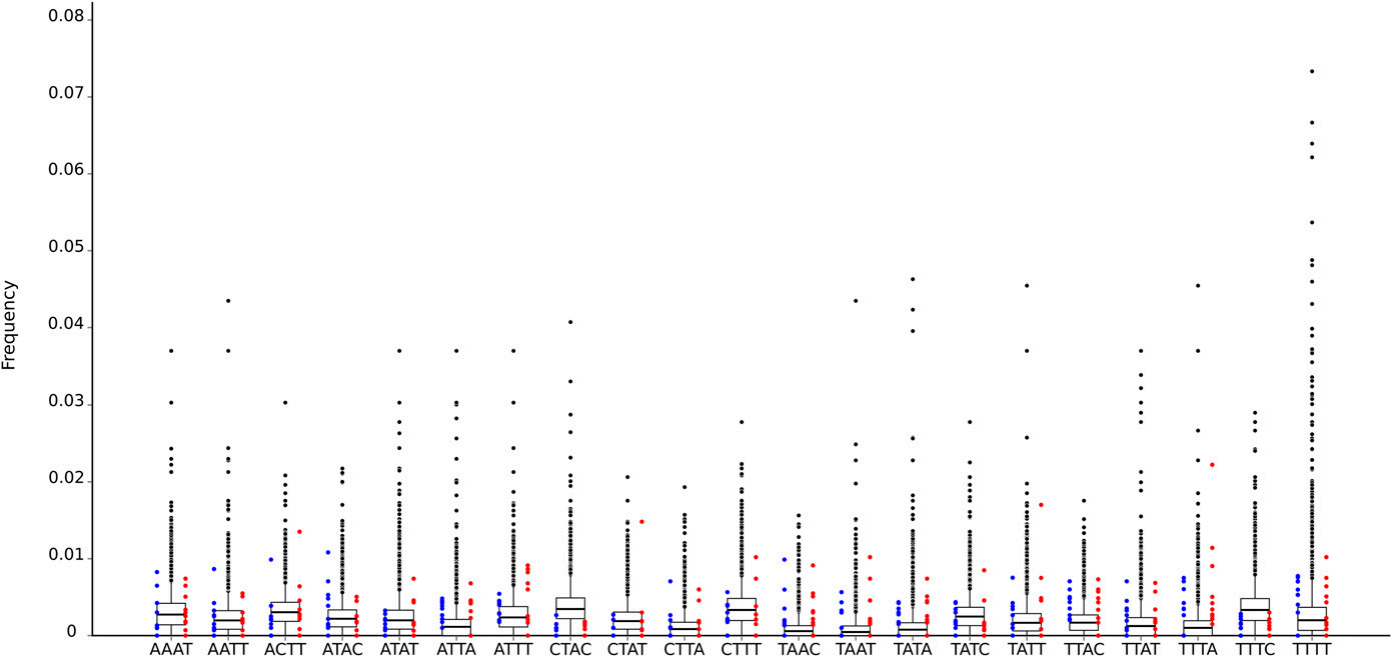
Frequencies of the most informative tetranucleotides selected by SigHunt in *E*. *festucae* genes. The most informative tetranucleotides selected by SigHunt are presented on the abscissa axis. The box plots represent the distribution of the number of each tetranucleotide found in *E. festucae* genes divided by the gene length. Blue dots on the left of each box plot represent the values for 10 genes randomly chosen from the closely related species *F. graminearum*, the red dots on the right to the values for 10 randomly chosen genes from a distantly related species *L. bicolor*.

More recent composition-based methods are based on tetranucleotide frequencies. For example, GOHTAM (Menigaud *et al.* 2012) and SigHunt (Jaron *et al.* 2014) build tetranucleotide signatures of species by computing the frequencies of all possible combinations of four nucleotides along a sliding window. The signatures are then used to find portions of the genomes that differ significantly from the average signature of the genome. SigHunt was used to extract those tetranucleotides with sufficient information to discriminate the *E. festucae* genome from its closest neighbor in our phylogenetic tree, *F. graminearum*. The distribution of the gene content for these tetranucleotides is presented in Figure 3. To simulate LGT events into *E. festucae*, 10 genes were randomly drawn from *F. graminearum* and *L. bicolor*, and overlaid on the distribution of *E. festucae* genes. Although the tetranucleotides were selected specifically to separate the two closest species, the tetranucleotide distributions clearly overlap between these three species, even with *L. bicolor*, the most distant species. Using these tetranucleotides, SigHunt identifies regions of the *E. festucae* genome that differ significantly in composition from the rest. These regions appeared to be picked primarily on their AT-rich status (Figure S1 in File S1). The genes that were included in the regions detected by SigHunt, which were mostly gene-poor isochores, were manually verified by looking at transcriptomic data (Eaton *et al.* 2015) to verify the gene model, and using blast results on the nr database of NCBI to check whether any evidence of LGT from a distant species could be found. No confirmation of LGT could be made and orthologs of all genes identified by SigHunt were found in closely related species. The gene *fitD* does not fall within a region detected by SigHunt.

A set of tetranucleotides displaying low variance among all genes in *E. festucae* was also computed (Figure S2 in File S1). LGT events might be observable as outliers for these low variance tetranucleotides. For example, it might be expected that *fitD* (Figure S2C in File S1) would display quite different values to housekeeping genes such as *tefA* (Figure S2B in File S1). However, values for *tefA* were in fact more different from the genome-wide gene signature than those of most *E. festucae* genes, including *fitD*.

To obtain an unbiased classification of the genes based on their composition, a data mining approach on the results of all the composition-based methods described above was performed. Transcript sequences of *E. festucae* were clustered using the Mean Shift clustering method (Comaniciu and Meer 2002) based on different measures of nucleotide composition, namely their CAI, ENC, GC, GC3, and trinucleotide (codon) and tetranucleotide frequencies. The automatically predicted bandwidth parameter placed all genes within a single cluster, suggesting that there are no genes whose composition differs significantly from any other genes in this dataset. Using the DBSCAN (Ester *et al.* 1996) clustering algorithm, the same phenomenon was observed. This indicates that these metrics appear to be insufficient to separate genes into different groups, and it follows that they are therefore incapable of discriminating laterally transferred genes from nonlaterally transferred genes.

**Figure 4:**
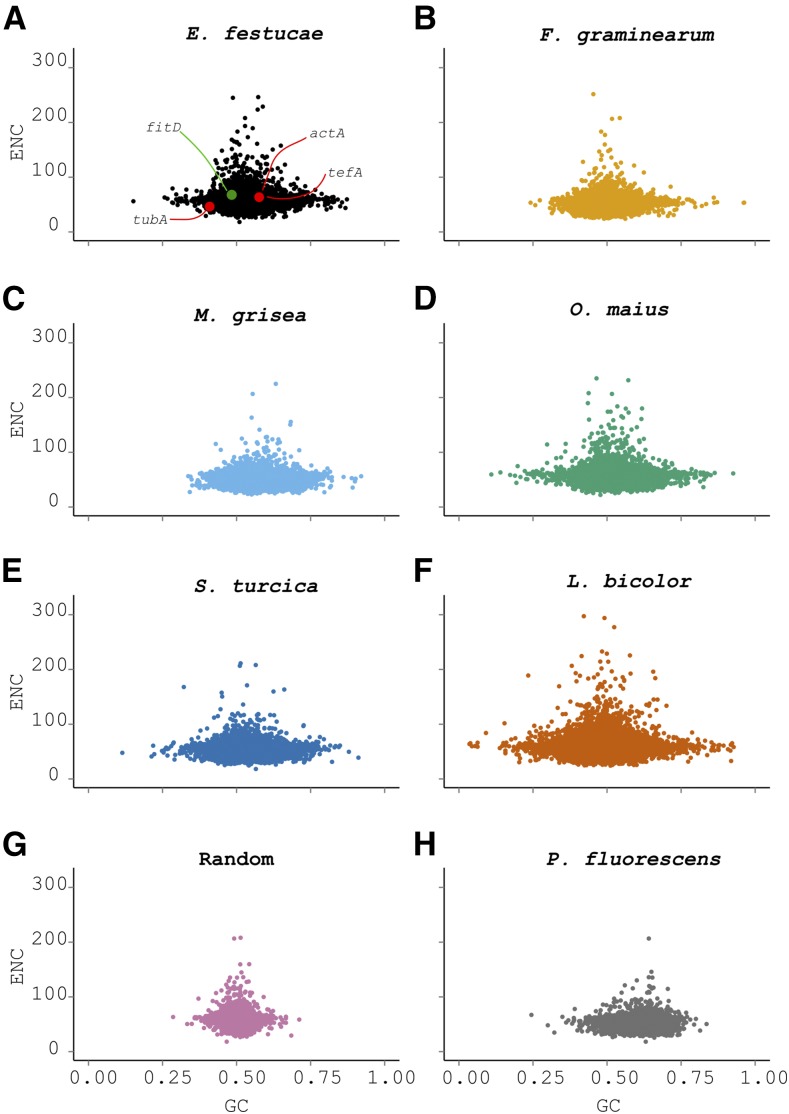
Comparison of coding sequence compositions (ENC and GC) for species at laddered evolutionary distances from *E. festucae*. Scatterplots comparing GC content to ENC. All abscissa and ordinate axes are plotted using the same scales. (A) Comparison of GC and ENC for the reference species *E. festucae*. Housekeeping genes tubA, actA, and tefA are highlighted in red (actA and tefA points overlap), while the fitD putative laterally transferred gene from a *Pseudomonas* bacterium is shown in green. The fungal species *F. graminearum* (B), *M. oryzae* (C), *O. maius* (D), *S. turcica* (E), and *L. bicolor* (F) show a marked overlap with the *E. festucae* data. (G) Sequences with the same size as *E. festucae* coding sequences, starting with ATG and ending with the same stop codon, but otherwise containing random nucleotides are shown. For comparison, the sequence composition of the bacterium *Ps*. *fluorescens* is also presented in (H). actA, actin; ENC, effective number of codons; fitD, cytotoxin FitD; GC, GC content; tefA, translational elongation factor 1α; tubA, α-tubulin gene.

Given the apparent failure of composition-based methods to identify laterally transferred genes in the *E. festucae* genome, values for the metrics used above (GC, GC3, CAI, and ENC) were compared across the five secondary fungal reference genomes indicated by stars on Figure 1, together with the genome of the bacterium *Ps. fluorescens*, which is a suspected source of the *fitD* potential LGT. To detect LGT, a method must be able to clearly discriminate between genes of different species. Scatterplots of GC *vs.* ENC for the five additional fungal reference species, together with *E. festucae* and *Ps. fluorescens*, are presented in Figure 4. These two metrics were chosen because they show the least overlap of all metrics used (Figure S3 in File S1 and Table 1). For *E. festucae*, the three housekeeping genes (*actA*, *tubA*, and *tefA*) and *fitD* all fall well within the cloud of points. Additionally, the distributions for the different fungal species, and even more surprisingly *Ps. fluorescens*, all overlap, indicating that it is not possible to discriminate between genes of these different species using these composition-based metrics. To further test the power of these metrics for species discrimination, sequences with the same size distribution as *E. festucae* coding sequences, but with random nucleotide content (although retaining the same start and stop codons), were generated and their content compared to the different species (Figure 4G). Once more, the distributions were all found to overlap. Figure S3 in File S1 and Table 1 summarize the overlap between composition values for the five fungal species, *Ps. fluorescens*, and the random sequences, with *E. festucae* values. Aside from the random sequences, the lowest overlap is seen between the GC content of *E. festucae* and *Ps. fluorescens*. This is expected since the bacterium *Ps. fluorescens* is the most phylogenetically distant species used in this study. If the data followed the known species phylogeny, the overlap should decrease from *F. graminearum*, the most closely related fungal species to *E. festucae*, to *L. bicolor*, the least closely related species (Figure 1). However, this is not the case (Figure S3 in File S1). For GC content, the greatest overlap is seen for *S. turcica*, and none of the metrics show a general decrease with increasing phylogenetic distance. This again questions the statistical power of these composition-based methods for detecting LGT events.

**Table 1:** Percentage overlap of distributions of gene composition metrics between *E. festucae* and other species, ordered by phylogenetic distance from *E. festucae*

	GC	GC3	CAI	ENC
*F*. *graminearum*	71.7	87.9	93.5	59.2
*M*. *oryzae*	70.3	55.2	78.7	76.2
*O*. *maius*	69.5	90.5	95.4	72.5
*S*. *turcica*	94.0	62.7	80.8	63.5
*L*. *bicolor*	63.5	95.5	97.6	35.6
*Ps*. *fluorescens*	40.7	56.1	75.4	61.7
Random	66.0	80.9	90.3	33.3
Median	69.9	75.3	87.2	62.6

The overlap in distributions of GC, GC3, CAI, and ENC is shown for *E. festucae* with five fungal species (*F. graminearum*, *M. oryzae*, *O. maius*, *S. turcica*, and *L. bicolor*) and a bacterium (*Ps. fluorescens*). Overlap with random sequences is also shown, although the median excludes these values. GC, GC content; GC3, GC content on the third position of the codons; CAI, codon adaptation index; ENC, effective number of codons.

### Phylogeny-based methods

**Figure 5:**
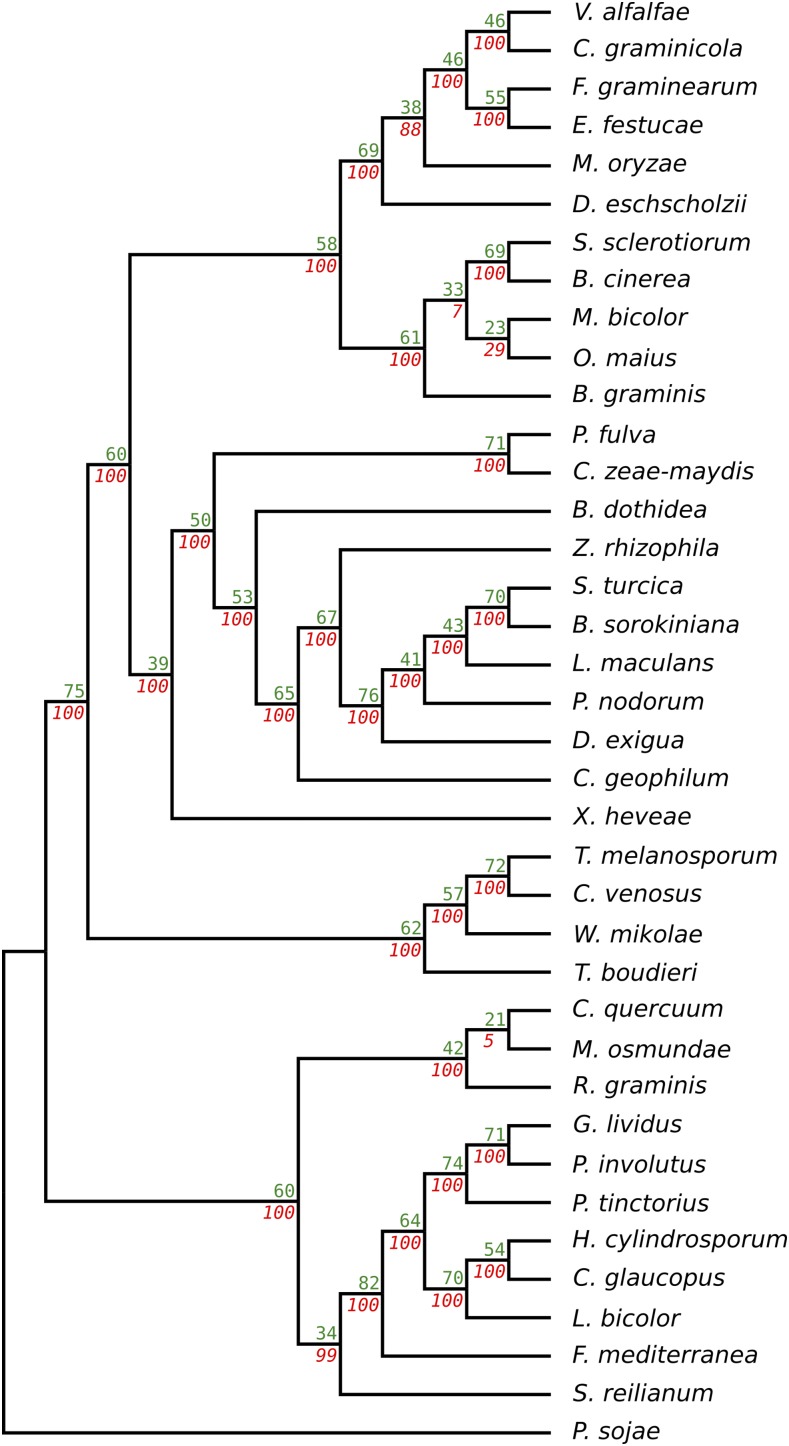
Statistical support for the internal nodes of the species tree. The taxa in this species tree are the same as those presented in Figure 1 and are given in the same order. The numbers indicated over the branches (green) show the percentage of the bipartitions in the gene trees for the node to the right of the number. The numbers under the branches (red) show the corresponding bootstrap values. All trees were rooted using the oomycete outgroup *P. sojae*.

A representative species tree is essential for any LGT detection method based on variation within the phylogeny. In this study, a species phylogeny was built using all gene trees containing a sequence from outgroup *P. sojae* (1768 trees). An SPR supertree was then built using only bipartitions with a bootstrap value ≥75% to increase the reliability of the final tree topology (Salichos and Rokas 2013). The generated tree (Figure 1) matches previously published fungal phylogenies (Ebersberger *et al.* 2012; Fitzpatrick *et al.* 2006; Wang *et al.* 2009) and fits with the traditional fungal classification in the NCBI taxonomy database (Sayers *et al.* 2009). The statistical significance of the branches in the species tree was computed as described in the *Methods*, and the resulting bootstrap values are presented under the branches in Figure 5. Most branches (88%) have bootstrap values >95%, showing strong support for most branches in the proposed species tree. Lower bootstrap values are observed in some small groups close to the tips of the tree, but all deeper groupings are supported by high bootstrap values.

**Figure 6:**
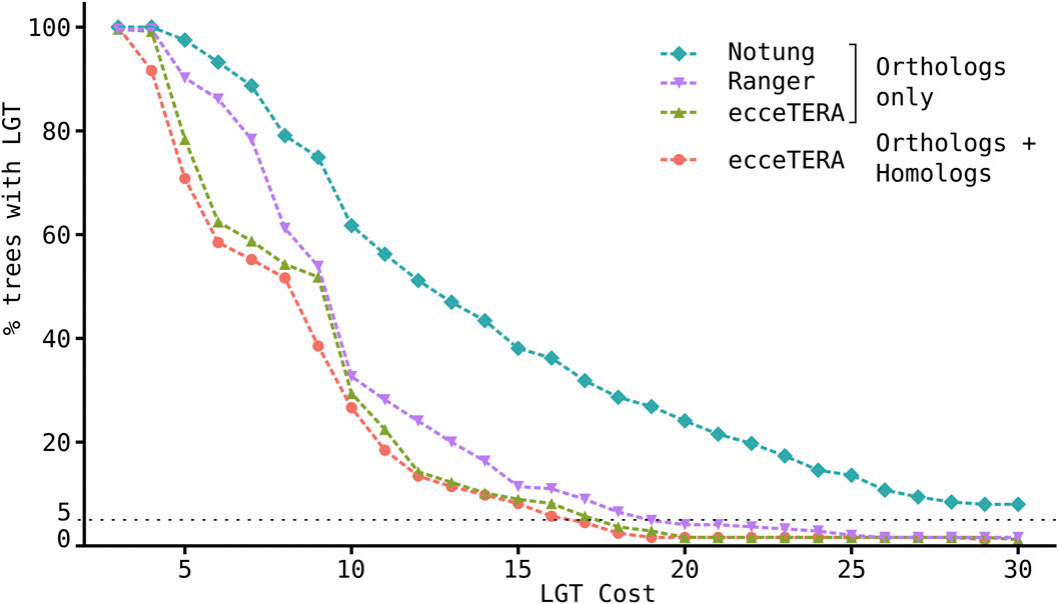
Variation in the number of lateral gene transfer (LGT) events predicted depending on the transfer cost in phylogeny-based LGT prediction methods. The percentage of trees displaying at least one LGT event predicted for transfer costs varying between 3 (the default cost) and 30 is shown for three different methods: Notung, Ranger-DTL, and ecceTERA. Only the gene trees with the best reciprocal blast orthologs were used for Ranger-DTL and Notung. Trees with homologs only, or homologs and orthologs combined (blast *E*-value < 1 × 10^−12^), are shown separately for ecceTERA. The theoretical rare event threshold of 5% is indicated by a black dashed line.

The SPRSupertrees (Whidden *et al.* 2014) software used to rebuild the species tree is also able to detect potential LGT events, but none were identified in this dataset. In contrast, Wisecaver *et al.* (2014) claim to have identified hundreds of LGT events in a fungal dataset of similar size. To see if the presence of potential paralogs in our dataset could affect the results of SPRSupertrees, other types of phylogeny-based LGT detection software, including programs that accommodate paralogs, were also applied to the dataset. Notung (Stolzer *et al.* 2012; Chen *et al.* 2000) and Ranger-DTL (Bansal *et al.* 2012) use a parsimony analysis to infer duplications, losses, and transfers of genes within a phylogenetic tree. Using the default costs recommended by their authors (LGT score = 3), both methods suggested that >99% of gene trees in the dataset contain at least one LGT event, and even when the cost of an LGT event was doubled, >50% of the gene trees were predicted to contain at least one LGT event. These results contrast strongly with the LGT detection results of SPRSupertrees and differ markedly with the general consensus that LGTs are relatively rare events. Published comparisons of the results from Notung and Ranger-DTL show that phylogeny-based methods for detecting LGT events often give high rates of false-positive results (Poptsova and Gogarten 2007; Nguyen *et al.* 2012). The software ecceTERA (Jacox *et al.* 2016) includes a gene tree correction method that aims to reduce the number of false-positives. EcceTERA results on trees containing only best reciprocal blast orthologs were compared to trees containing all homologs based on a blast *E*-value < 1 × 10^−12^, but the results returned were comparable in both cases (Figure 6).

By definition, an LGT event will create a discrepancy between the gene and species trees. Likelihood-based methods can be used to detect such differences. It is possible to constrain the gene tree topology by the species tree topology, and compute the likelihood of the constrained gene tree by letting PhyML optimize only the branch lengths and the rate parameters, but not the topology. By doing this, the final phylogenetic tree will have the same topology as the species tree, only the branches will have different lengths. In the absence of any LGT event, the gene tree would be expected to be close to the species tree, so constraining the gene tree using the species tree topology should have little impact on its likelihood. However, in the presence of an LGT event from a species included in the tree (or a close relative from outside the species set), the length of the branch carrying the LGT event will significantly increase, resulting in a gene tree with lower likelihood. The likelihoods of the constrained and unconstrained trees, both built using the same model, are then compared. The AIC (Akaike Information Criterion), which can be computed on nonnested models, was then calculated from the two likelihoods to verify whether they are meaningfully different. For this comparison, the degrees of freedom correspond to the number of internal branches on the tree. No significant difference between the constrained and unconstrained gene trees was observed; in other words, no LGT events were again detected. To identify whether this method is really capable of detecting LGTs, computer simulations of LGT events were performed. Specifically, random genes were computationally transferred from *L. bicolor*, *S. turcica*, *M. oryzae*, and *O. maius* to *E. festucae*. The gene tree topologies were again constrained using the species tree, and the AIC of the constrained and unconstrained trees computed. Again, no significant differences were found, likely reflecting the high number of degrees of freedom that causes an increase in the significance threshold. In practice, this method, which is conceptually a cornerstone of many phylogenetic-based LGT detection techniques, appears to have low statistical power to detect LGT events.

**Figure 7:**
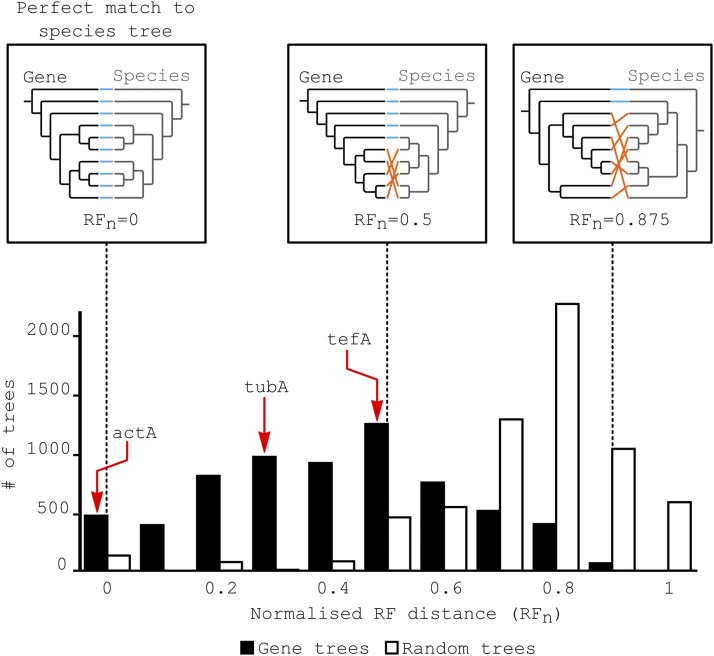
Variation in the topology of gene trees compared to the species tree. The distribution of the RFns between the species tree and between the species tree and trees of individual *E. festucae* genes is shown in black. The distribution of the RFn distances between the species tree and randomly generated trees with the same size distribution as the real gene trees is displayed in white. The distance values for the housekeeping genes actA, tubA, and tefA are highlighted by red arrows. The fitD gene cannot be represented on this figure because it is only present in *E. festucae*. (This putative laterally transferred gene is not found in the other fungi studied here.) Examples of comparisons between gene trees and the species tree are displayed in the boxes at the top part of the figure. The species tree appears to vary only because the subset of species present in the gene tree differs between the three examples. The species tree topology is in fact identical for all analyses. Orange links indicate a change in the location of species in the gene tree relative to the species tree. Blue links indicate conservation with the species tree topology. RFn = 0 corresponds to a perfect match between the species tree and the gene tree, while RFn = 0.5 indicates that half of the nodes in the gene tree do not match the species tree. actA, actin; fitD, cytotoxin FitD; RFn, normalized Robinson and Foulds distances; tefA, translational elongation factor 1α; tubA, α-tubulin gene.

Even though the SPR supertree was identical to that expected based on the published literature, substantial variation in the topologies of the gene trees was observed relative to the final species tree. This has also been noted by Salichos and Rokas (2013), and is reflected by the low proportion of species tree bipartitions (nodes of the tree) present in the gene trees (Figure 5). Indeed, 31% of the nodes in the supertree are present in fewer than half of the gene trees, and only 34% are present in at least two-thirds of the gene trees. To determine how topologically distant the gene trees are from the species tree, the normalized RF topologic distance (RFn) (Robinson and Foulds 1981) between each gene tree and the species tree was computed. The distribution of these distances is presented in Figure 7. Contrary to expectations, most of the distances between the gene trees and the species tree are not close to zero, meaning that some of the gene trees have very different topologies from the species tree. To verify whether the observed distribution might simply be due to randomness, possibly resulting from incorrect orthology calls or problems with small numbers of taxa in some of the gene trees, the leaves of each gene tree were shuffled to create random trees with the same size distribution as the original set of trees (Figure 7). As the distributions of the real and random data are different, it is unlikely that the distances of the gene trees to the species tree are solely due to a gene tree size effect, or to random effects, at least across the entire tree. It was particularly surprising to observe that even housekeeping genes like *tubA* (RFn = 0.33) and *tefA* (RFn = 0.5) display large topological distances from the species tree.

## Discussion

LGT is a “hot topic” in molecular biology (Soucy *et al.* 2015) and has almost become a default explanation for genes that do not display an expected phylogenetic distribution or composition. However, identification of LGT events is no trivial exercise (Ragan 2001; Morel *et al.* 2015; Ovadia *et al.* 2011) and detection methods are expected to be highly dependent on the quality of the underlying genomic data. Recently, many LGT events in eukaryotic species have been called into question (Danchin 2016; Ku *et al.* 2015).

In this study, we assess the reliability of automated LGT detection methods for eukaryotes using datasets derived from plant-associated fungi, as a counterpoint to the extensive analyses performed on bacterial systems (Lawrence and Ochman 1997; Garcia-Vallve *et al.* 2000; Nakamura *et al.* 2004). Both composition- and phylogeny-based approaches were assessed for their ability to detect both putative and simulated LGT events in a eukaryotic fungal dataset. This included *fitD*, a gene originally described as laterally transferred from *Pseudomonas* bacteria into *Epichloë* endophytes (Ambrose *et al.* 2014), but as it is also present in many other fungi, its LGT status remains uncertain.

Given the wealth of genomic data now available, it is impractical to manually verify all protein or gene models, orthology predictions, or multiple sequence alignments, requiring researchers to accept some margin of error and level of mistakes in their data. It is difficult to predict *a priori* the extent to which these problems affect the bioinformatics tools and techniques used in LGT detection. For this reason, our analysis was purposely performed using typical uncurated genomic datasets, so as to mimic as closely as possible the situation encountered by most users of the various LGT prediction tools.

Composition-based methods are, in theory, ideal for predicting LGT events; they do not require any knowledge about the studied organism other than its genomic sequence, and because they do not rely on phylogenetics, they are generally much faster to use. Moreover, no information about other organisms is needed for comparison and knowledge of the source species of the LGT event is not required. However, it is important to acknowledge that, strictly speaking, composition-based methods do not detect LGT events, but rather show genes whose composition differs significantly from the rest of the genome. Nonetheless, LGT is not the only explanation for differences in gene composition, as this can also be caused by common non-LGT genetic processes, such as rapid gene evolution, pseudogenization, or simply being located close to AT-rich isochores. Therefore, not all genes that display unexpected composition patterns necessarily result from LGT events. Composition-based detection methods have been evaluated in bacterial genomes (Cortez *et al.* 2005) by studying the ability of different methods to detect artificial gene transfers to *E. coli* K12 MG1655 from different proteobacteria and prokaryotes. This *E. coli* study revealed the highly variable rates of efficacy of these methods, especially for the CAI/GC content metric, with detection of LGT events ranging from 6 to 92%. Here, four gene composition metrics were assessed for their reliability in detecting potential LGT events in fungi: GC, GC3, CAI, and ENC. Surprisingly, none of these metrics were able to separate random sequences from true genes, showing that these methods have no power to detect LGT events in our dataset. Manual analysis of genes at the extremes of the four metric distributions (2.5% tails), where laterally transferred genes would be expected to fall, failed to reveal any obvious signs of LGT based on homology searches with other species. Inclusion of a fifth metric, the more recently developed tetranucleotide frequencies, identifies AT-rich isochores as LGT events, showing that this method cannot be applied to fungal genomes without preliminary filtering of AT-rich regions, repetitive elements, and probably other genomic features like pathogenicity islands. Even the most informative tetranucleotide identified by SigHunt failed to separate closely related *F. graminearum* and distantly related *L. bicolor* genes from *E. festucae* orthologs (Figure 3).

A crucial assumption in the use of composition-based methods for LGT detection is that gene composition metrics vary between species, and this variation is presumed to increase with phylogenetic distance. Here, we show that this is not true. GC and ENC displayed the lowest average overlap between the different species, but even when these two metrics were combined in a two-dimensional comparison, no significant differences were observed, even against bacterial genes and random sequences. This inability to separate data derived from different species, or even random data, questions the usefulness of these metrics for LGT detection. These metrics have been widely used for predicting LGT events in prokaryotes (Gogarten and Townsend 2005; Keeling and Palmer 2008; Shapiro 1969; Gyles and Boerlin 2014; Kay *et al.* 2002; Furuya and Lowy 2006; Ochman *et al.* 2000; Martínez 2008), and this study does not question those findings. Rather, we query the widely-assumed suitability of these metrics for analyzing eukaryotic data. Eukaryote gene structures are more complex than in prokaryotes, with the presence of introns and exons that are often poorly annotated in nonmodel organisms. LGT prediction may be even more complex in fungi because they display rapid evolution, especially in plant-associated fungi (Huang *et al.* 2014; Le Quere *et al.* 2006; Brundrett 2002; Terhorst *et al.* 2014), and particularly for genes involved in pathogenicity or symbiosis. Therefore, it might be expected that laterally transferred genes would evolve rapidly to display a similar composition to the rest of the genome.

This study also evaluated the reliability of phylogeny-based methods for LGT detection, using simulated LGT events into *E. festucae* as a benchmark for detection. Phylogeny-based methods are generally considered to be more reliable than composition-based methods, especially for detecting transfers between closely related species. However, there are considerable inconsistencies between the results of different detection methods (Ragan 2001; Jaron *et al.* 2014). Additionally, phylogeny-based methods seem to be very sensitive to the quality of the study data (Wägele and Mayer 2007) and it was unclear how these methods would perform on real uncurated datasets, particularly those representing larger eukaryotic genomes. Phylogeny-based methods work by comparing the topology of the species tree with topologies for gene trees to identify genes that display a significantly different phylogeny, thus hinting at possible LGT events.

The species supertree was built so as to minimize the distance between all gene trees. Due to the nature of the resampling process used to compute the bootstrap scores, each bootstrap replicate tends to be based on a very large dataset (despite the resampling) and thus all bootstrap trees are very similar to the species supertree. Consequently, bootstrap scores are typically close to 100%. This seems overly conservative and it proved more informative to look at the distribution of tree shapes rather than the bootstrap values. To check whether high bootstrap values are an artifact of the large set of gene trees, the frequencies of the species tree bipartitions present across all the gene trees were computed. This provides a measure of the variation in the underlying gene phylogenies. Comparing individual gene tree topologies with the species supertree revealed a surprisingly high level of variation among individual gene trees, with the frequency of bipartition identities ranging from 21 to 82%, with an average of 56%. This means that a given node in the species tree is on average present only in around half of the gene trees. This was unexpected as it is generally thought that individual gene trees mostly follow the same phylogeny as the species tree, although this marked variation between gene trees and the species tree topology has been observed previously in bacteria (Poptsova and Gogarten 2007) and yeasts (Salichos and Rokas 2013). It is likely that the low bipartition identities observed are at least in part due to pruning of nodes in the gene trees with bootstrap values <75%. However, this was essential to ensure that only highly supported nodes were used for the phylogeny comparisons. It is also interesting to note that low bootstrap values were always associated with a low bipartition frequency value, but that the reciprocal property was not true: some high bootstrap values were associated with a low bipartition frequency. For example, the node that separates the Xylonomycetes and Dothideomycetes has a bootstrap score of 100%, but a bipartition frequency score of only 39%. The fact that these frequencies are generally low emphasizes just how variable gene tree topologies are. As phylogeny-based LGT methods work by identifying gene phylogenies that differ from that of the species tree, such extreme levels of topological variation between the gene trees and species tree poses a considerable problem for these detection methods, as it then becomes complex to distinguish LGT events from normal topological variation.

Phylogeny-based methods compare gene and species trees by computing a distance value between them. In this study, the high variation between the gene and species trees by definition also impacts the RFn, where a distance of zero represents an identical topology for a given gene tree and the species trees, while a distance of one corresponds to the two trees being as different from each other as possible. It was expected that most of the gene tree topologies would reflect the species tree topology and have distances very close to zero. In this case, the distribution of the topological distances between the species tree and gene trees would be represented by a rapidly decreasing curve with most of the distances close to zero. LGT events should then be represented by gene trees with higher distances. Instead, a bell-shaped distribution was observed, centered at ∼0.5 and with a maximum of 0.9, indicating that most of the gene trees are very different from the species tree. This implies that, although the species tree is the average of all the gene trees, it is by no means the most frequent tree. In fact, only 6% of gene trees exactly match the species tree. The sheer diversity of gene tree topologies may be due to the variable speed of gene evolution resulting from quite different environmental selection pressures or incomplete lineage sorting. It is generally thought that discordant gene and species phylogenies reflect only rare events like LGT or transspecies polymorphism (Pamilo and Nei 1988; Maddison 1997), and many putative laterally transferred genes have been identified based on this assumption. However, this study reveals that, at least in fungi, discord between gene and species tree topologies appears to be common and can even be observed routinely in housekeeping genes. We suspect that this large variation in tree topologies is the main reason why the SPRSupertrees software could not detect any LGT events in this dataset, and may also explain the unlikely results of Notung and Ranger-DTL, which predict that nearly all gene trees contain at least one LGT event. These tools apply a score for each type of event (duplications, gene losses, and LGTs), and the software returns the most parsimonious series of events relative to the phylogeny. Without *a priori* knowledge of the rates at which these events occur, it is challenging to find an appropriate set of scores for the algorithm. In this study, the default scores produce unlikely results. Moreover, missing genes in our gene trees may have important consequences: they should be annotated as losses, but often seem to be classified as transfers. EcceTERA attempts to address this by implementing a probabilistic model to infer true transfer events from false-positives. The relationship between the number of detected LGT events and the transfer cost in Notung, Ranger-DTL, and ecceTERA are inverse-like functions; doubling the default score for a transfer event still results in >50% of the trees displaying an LGT event. Even when the default transfer cost is multiplied by five, LGT events still are still not rare, with >5% of trees (8 to 38% of trees) being called as having at least one LGT event. This illustrates how difficult it can be to choose a transfer cost for these methods and how unreliable the default scores can be.

Often, missing orthologs and discontinuous distributions of homologs are also used to infer potential LGT events (Ambrose *et al.* 2014; Moore *et al.* 2014; Fitzpatrick *et al.* 2008; Temporini and Van Etten 2004), but these assumptions tend to change as more genomes are sequenced and intermediate genes are found (Khaldi *et al.* 2008). Finally, many of the tools developed to detect LGT events are based on phylogenetic methods that have been developed for bacteria, but in practice, fungal phylogenies are much harder to reconstruct than bacterial phylogenies due to the presence of cryptic intronic sequences and other sequence complexities. Additionally, the genomes of plant-associated fungi often contain effector genes, which are short and highly variable (Mesarich *et al.* 2015), as well as genes involved in the production of secondary metabolites, which tend to evolve rapidly and often display a complex modular structure (Berry *et al.* 2015). The phylogeny is particularly hard to reconstruct for these genes. Thus, the efficacy of LGT detection tools in fungi, and presumably for other eukaryotes, seems to remain relatively underpowered. In addition to the deficiencies mentioned above, the phylogeny-based LGT detection methods also completely failed to identify either the putative LGT event of *fitD*, any other potentially novel LGT events, or any computationally simulated LGT events. This leads us to express doubt regarding the overall reliability of these tools for LGT detection in similar eukaryotic datasets. Indeed, this study highlights the key problem of using incompletely annotated genome sequence information for automated LGT prediction. Where orthologs could not be identified in some species, this may not have been due to the absence of these genes in those species, but rather from the sequence simply being missing from the dataset. As missing taxa in gene trees are a potential indicator of LGT (Temporini and Van Etten 2004; Fitzpatrick *et al.* 2008), this problem would also confound LGT detection methods.

Overall, this study reveals concerning insight into the deficiencies of both composition- and phylogeny-based LGT detection methods when used on complex, uncurated, but typical eukaryotic genome sequence data. Neither of the broad approaches was capable of identifying a putative LGT event or gave reliable support for any novel LGT event. This is in striking contrast to the large number of claims that routinely appear in the literature. Additionally, this study exposes the existence of extremely high variation between gene tree and species tree topologies across fungi, showing that this major biological pattern holds beyond the yeasts. An important effect is that this variation will likely confound phylogeny-based LGT prediction methods. This study concludes that LGT remains a challenging process to detect, and questions whether the flood of LGT events appearing in the literature to date are necessarily well supported statistically.

## Supplementary Material

Supplemental material is available online at www.g3journal.org/lookup/suppl/doi:10.1534/g3.116.038448/-/DC1.
