# Supplementary material for: Genomic Data Quality Impacts Automated Detection of Lateral Gene Transfer in Fungi
Source: G3 (Bethesda). 2017 Feb 23;7(4):1301–14. doi: 10.1534/g3.116.038448 (PMC5386878; doi:10.1534/g3.116.038448)
Supplement: Supplementary file 1 [file 1301FileS1.docx]

# Figures


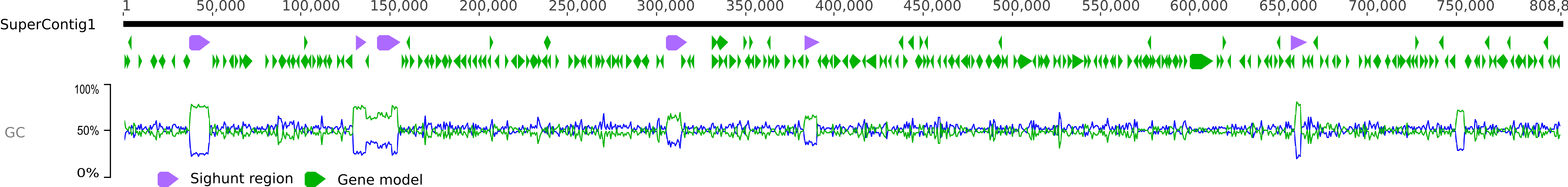


Figure S1 – Prediction of regions with a significantly different composition in the Epichloë festucae longest supercontig as determined by SigHunt

The regions with significantly different composition as predicted by SigHunt are displayed in purple. Gene models are shown in green. The AT and GC percentages are displayed in green and blue respectively on the bottom track. The SigHunt predictions fall into the AT rich isochores of the Epichloë festucae genome and are not associated with genes.


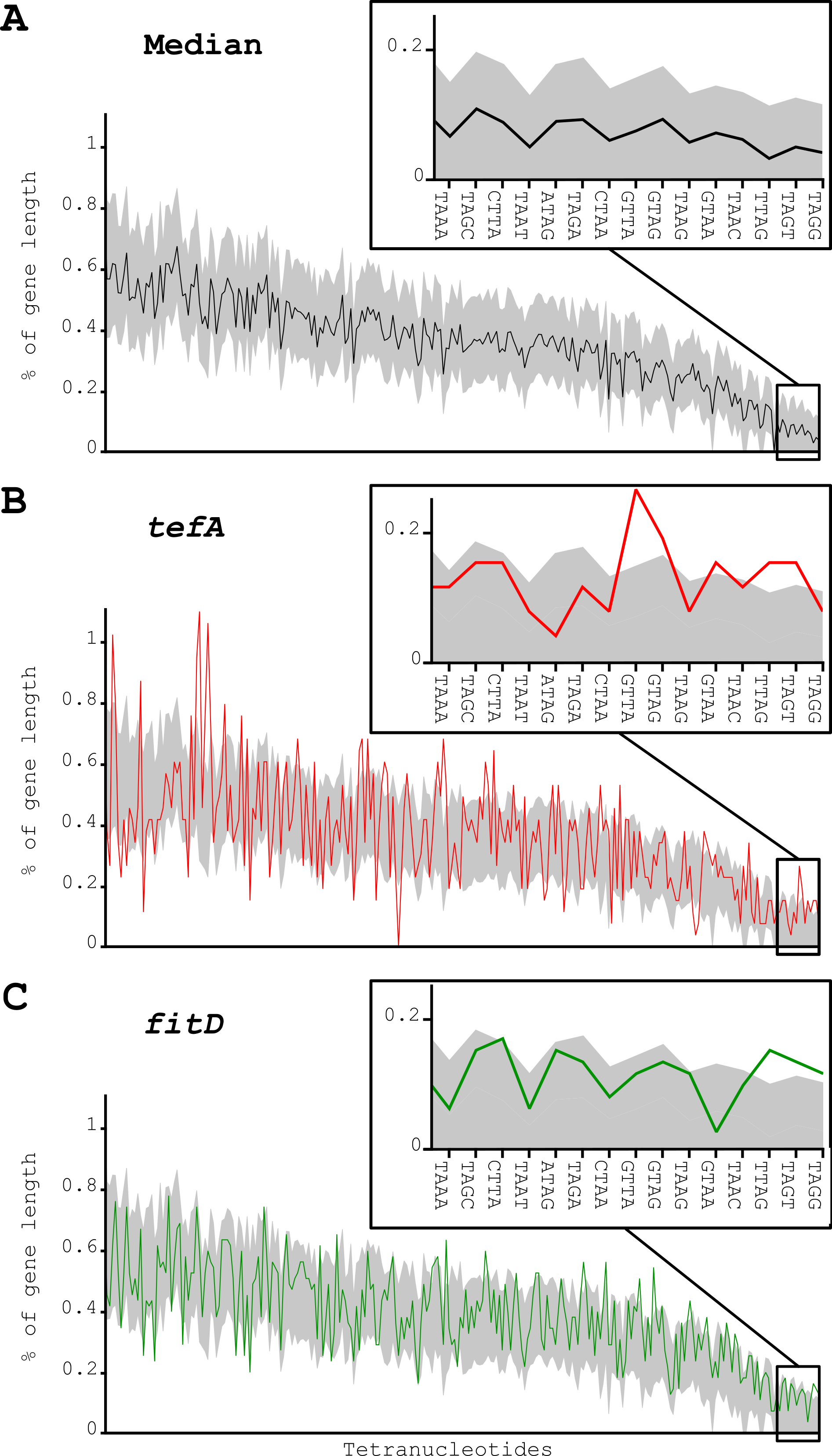
Figure S2 - Distribution of tetranucleotide frequencies in Epichloë festucae coding sequences.

The tetranucleotides are presented on the abscissa axis. The percentage of the length of the coding sequences corresponding to each tetranucleotide is presented on the ordinate axis. The interquartile range of each tetranucleotide on all genes is shown in grey (same in all subfigures). Tetranucleotides are sorted by decreasing variance. A zoom of the last fifteen tetranucleotides is presented in the top right corner of each subfigure. A: median of the percentage of the gene length for each tetranucleotide. B: tetranucleotide values for E. festucae tefA (translation elongation factor 1 alpha) gene in red. This is a housekeeping gene used as a reference for a non laterally transferred gene. The grey shading corresponds to the general interquartile range of all E. festucae genes (as in A). C: tetranucleotide values for fitD (cytotoxin FitD), a putative laterally transferred gene from a Pseudomonas bacterium, in green. The grey shading again corresponds to the general interquartile range of all E. festucae genes.


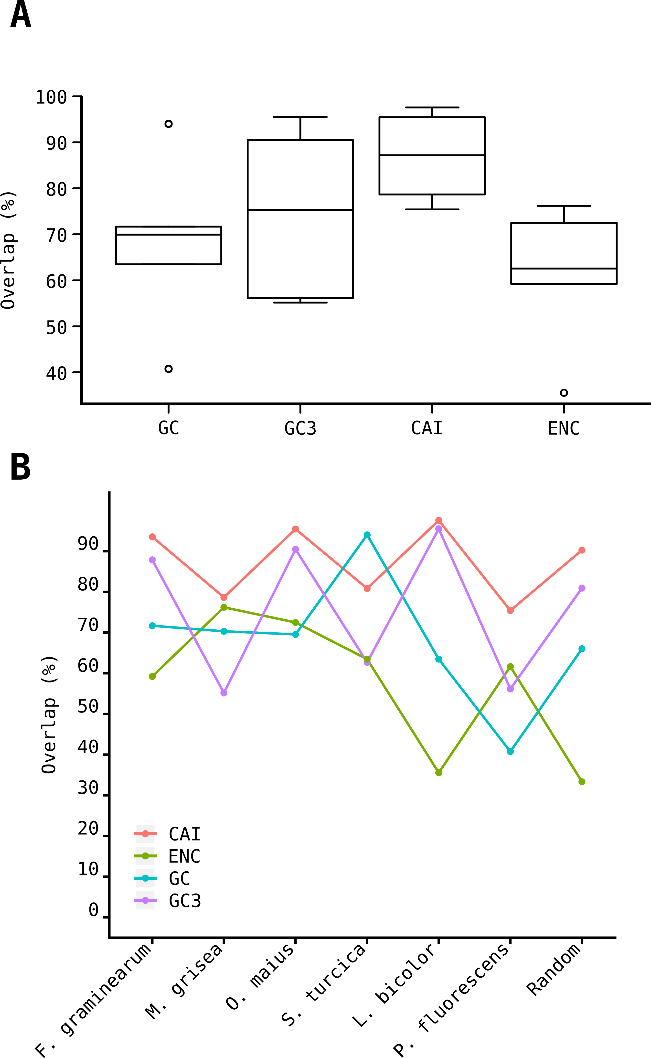


Figure S3 – Overlap of gene composition metrics between Epichloë festucae and other species at sequential evolutionary distances.

The metrics are: GC (GC content), GC3 (GC content on the third base of the codons), CAI (Codon Adaptation Index) and ENC (Effective Number of Codons). The species are the fungi F. graminearum, M. grisea, O. maius, S. turcica and L. bicolor and the bacterium P. fluorescens. The random sequences present in B correspond to random sequences with the same size as E. festucae coding sequences, starting with an ATG codon and ending with a stop codon. A: boxplots of the overlap with E. festucae for each metric for all species (excluding the random sequences). B: Scatterplot of the average values of the overlap for each of the metrics per species. Note that the expected regular decline in overlap values with sequentially greater evolutionarily distance does not occur.

# Tables

| **Species** | **Strain** | **JGI id** |
| --- | --- | --- |
| *Daldinia eschscholtzii* | EC12 v1.0 | DalEC12_1 |
| *Rhodotorula graminis* | WP1 v1.0 | Rhoba1_1 |
| *Xylona heveae* | TC161 v1.0 | Xylhe1 |
| *Cenococcum geophilum* | 1.58 v1.0 | Cenge1 |
| *Choiromyces venosus* | 120613-1 v1.0 | Chove1 |
| *Cortinarius glaucopus* | AT 2004 276 v2.0 | Corgl3 |
| *Gyrodon lividus* | BX v1.0 | Gyrli1 |
| *Hebeloma cylindrosporum* | h7 v2.0 | Hebcy2 |
| *Laccaria bicolor* | S238N-H82 v2.0 | Lacbi2 |
| *Meliniomyces bicolor* | E v1.0 | Melbi2 |
| *Oidiodendron maius* | Zn v1.0 | Oidma1 |
| *Paxillus involutus* | ATCC 200175 v1.0 | Paxin1 |
| *Pisolithus tinctorius* | Marx 270 v1.0 | Pisti1 |
| *Terfezia boudieri* | S1 v1.0 | Terbo1 |
| *Tuber melanosporum* | Mel28 v1.0 | Tubme1 |
| *Wilcoxina mikolae* | CBS 423.85 v1.0 | Wilmi1 |
| *Bipolaris sorokiniana* | ND90pr v1.0 | Cocsa1 |
| *Blumeria graminis* | f.sp. hordei DH14 | Bulgr1 |
| *Botryosphaeria dothidea* |  | Botdo1 |
| *Botrytis cinerea* | B05.10 v1.0 | Botci1 |
| *Cercospora zeae-maydis* | v1.0 | Cerzm1 |
| *Colletotrichum graminicola* | M1.001 | Colgr1 |
| *Cronartium quercuum* | G11 v1.0 | Croqu1 |
| *Didymella exigua* | CBS 183.55 v1.0 | Didex1 |
| *Fomitiporia mediterranea* | MF3/22 | Fomme1 |
| *Fusarium graminearum* | PH-1 (NRRL 31084) | Fusgr1 |
| *Leptosphaeria maculans* | JN3 | Lepmu1 |
| *Magnaporthe oryzae* | 70-15 (MG8) | Maggr1 |
| *Mixia osmundae* | IAM 14324 v1.0 | Mixos1 |
| *Passalora fulva* | 0WU CBS131901 | Clafu1 |
| *Phaeosphaeria nodorum* | SN15 v2.0 | Stano2 |
| *Sclerotinia sclerotiorum* | ATCC18683 | Sclsc1 |
| *Setosphaeria turcica* | Et28A v1.0 | Settu1 |
| *Sporisorium reilianum* | SRZ2 | Spore1 |
| *Verticillium alfalfae* | VaMs. 102. | Veral1 |
| *Zopfia rhizophila* | CBS 207.26 v1.0 | Zoprh1 |
| *Phytophthora sojae* | P6497 | Physo3 |
| *Pseudomonas fluorescens* | F113 |  |

Table S1 – Strain and JGI accession numbers of the plant-associated fungal genomes

|  | **Min** | **1^st^ quart.** | **Median** | **3^rd^ quart.** | **Max** | **IQR** | **CV** |
| --- | --- | --- | --- | --- | --- | --- | --- |
| **GC** | 0.15 | 0.50 | 0.53 | 0.57 | 0.87 | 0.07 | 0.11 |
| **GC3** | 0.12 | 0.55 | 0.61 | 0.68 | 0.96 | 0.13 | 0.16 |
| **CAI** | 0.63 | 0.73 | 0.76 | 0.78 | 1.00 | 0.05 | 0.05 |
| **ENC** | 18.20 | 50.51 | 56.51 | 60.13 | 208.10 | 9.62 | 0.17 |

Table S2 - Summary statistics for the distribution of composition metrics of E. festucae coding sequences.

Columns correspond to the minimum, first quartile, median (second quartile), third quartile, maximum, interquartile range and coefficient of variation (ratio of the standard deviation to the mean).
